# Erythrocyte Senescence in a Model of Rat Displaying Hutchinson-Gilford Progeria Syndrome

**DOI:** 10.1155/2018/5028925

**Published:** 2018-05-29

**Authors:** Manoj Kumar Chaudhary, Syed Ibrahim Rizvi

**Affiliations:** ^1^Department of Biochemistry, Janaki Medical College and Teaching Hospital, Ramdaiya Bhawadi, Dhanusha, Nepal; ^2^Department of Biochemistry, University of Allahabad, Allahabad 211002, India

## Abstract

**Background:**

Increased oxidative stress is a major cause of aging and age-related diseases. Erythrocytes serve as good model for aging studies. Dihydrotachysterol is known to induce premature aging feature in rats mimicking Hutchinson-Gilford progeria syndrome.

**Aim:**

In the present study, attempts have been made to explore the differential response of young and senescent erythrocytes separated by density gradient centrifugation from accelerated senescence model of rats mimicking Hutchinson-Gilford progeria syndrome and naturally aged rats.

**Methods:**

The erythrocytes of naturally aged and progeroid rats were separated into distinct, young and old cells on the basis of their differential densities. The parameters of oxidative stress and membrane transport systems were studied.

**Discussion and Conclusion:**

Our study provides evidence that organismal aging negatively affects oxidative stress markers and membrane transport systems in both young and old erythrocytes. This study further substantiates that the changes in progeria model of rats resemble natural aging in terms of erythrocyte senescence.

## 1. Introduction

Hutchinson-Gilford progeria syndrome (HGPS) is a sporadic disorder characterized by premature aging. The primary etiology of the disease is a genetic mutation wherein cytosine to thymine substitution at position 1824 of Lamin A (LMNA) gene located in exon 11 erroneously produces a mutant prelamin A with the deletion of 50 amino acids including proteolytic cleavage site required for its post translational maturation. This mutant prelamin A protein is called progerin which retains farnesyl group through which it remains anchored tightly to the nuclear envelope leading to their accumulation and consequent nucleo-skeleton defect resulting into features of aging [1, 2]. Progerin accumulation is not only confined to HGPS but also its progressive accumulation has been studied in relevance to normal aging [3, 4]. Importantly, progerin accumulation has been reported to promote oxidative stress [5].

It has been reported that chronic intoxication with dihydrotachysterol (DHT) to young rats induces premature aging mimicking HGPS-like syndrome [6]. This progeroid rat model has been reported to mimic natural aging in terms of impaired redox status [7]. Erythrocytes exhibit numerous senescence markers including microvesiculation, decreased cellular volume, increased cellular density, and modulation in membrane transport system, which appear progressively during their lifespan before they exit the circulation at the end of their life span [8]. Although the erythrocytes are enucleated cells without any biosynthetic machinery, they maintain their viability till the end of their life span and are widely considered as viable model to study the aging process [9, 10].

The erythrocyte membrane, enriched with polyunsaturated fatty acids, is a soft target of reactive oxygen species (ROS) which makes them susceptible towards oxidative damage and consequently early removal from circulation [11]. Recently, we have reported an altered redox balance in DHT-induced HGPS mimicking premature aging in rats [7]. In view of above, the present study was undertaken to correlate organismal aging with cellular senescence and its differential effect on young as well as old erythrocytes from naturally aged and HGPS mimicking accelerated aging model of rat. To meet the above objectives, the biomarkers of oxidative stress were evaluated in young, old, and unfractionated erythrocytes isolated from normal young, naturally aged and DHT-induced aged rats through density gradient centrifugation.

## 2. Materials and Methods

### 2.1. Reagents and Chemicals

Dihydrotachysterol (DHT), corn oil, DIDS (4,4-diisothiocyanatostilbene-2,20-disulphonic acid), amiloride hydrochloride, ouabain, 2,4,6-tri(2-pyridyl)-s-triazine (TPTZ), reduced glutathione (GSH), 4,7-diphenyl-1,10-phenanthroline disulfonic acid sodium salt (DPI), 5,5-dithiobis nitro benzoic acid (DTNB), and percoll were obtained from Sigma Aldrich, St. Louis, USA. The rest of the chemicals used were purchased from SRL and HIMEDIA Lab, India.

### 2.2. Animal Model and Experimental Protocol

A total of 24 female Wistar rats of different ages were used in the study. Female rats were preferentially used because a higher DHT dose is required to induce progeria-like syndrome in correspondingly aged male rats [12]. The rats were subjected to a week-long acclimation prior to start of dosing and were retained in polypropylene cages maintained at 25 ± 5°C (4rats/cage) temperature and 12/12 h light/dark periodic cycle. They were fed with standard laboratory pellet diet obtained from Paramount Techno Chem., Varanasi, India, with free access to drinking water. The total duration of the study was 30 days with the following treatment schedule.

- Group I: young control (3 months) group (*n* = 8) with body weight of 120 ± 20 g, receiving no treatment
- Group II: DHT treatment (3 months) group (*n* = 8) with body weight of 120 ± 20 g, receiving 50 *μ*g DHT dissolved in 0.5 mL corn oil, once daily through oral gavage route [6]
- Group III: old control (24 months) group (*n* = 8) with body weight of 350 ± 20 g, receiving no treatment

The protocols and guidelines laid by animal ethical committee of University of Allahabad were followed during the study.

### 2.3. Blood Collection Procedure and Separation of Red Blood Cells from Plasma

After completion of the treatment schedule, blood samples were drawn by cardiac puncture under light anesthesia and collected using heparin as anticoagulant. The blood samples were centrifuged at 800 ×g for 15 min at 4°C, and plasma was separated from packed red blood cells (PRBCs). After removal of the upper 15% buffy layer, PRBCs were washed thrice with cold phosphate-buffered saline (PBS) and finally suspended in glucose-containing PBS (GPBS) for use in biochemical analyses.

### 2.4. Density-Based Separation of Young and Old Erythrocytes and Isolation of Membrane Fraction

Erythrocytes of blood samples of different groups were separated into young and old populations on percoll-based density gradient centrifugation as described previously [13]. Briefly, the PRBCs were washed with RPMI 1640 medium twice followed by resuspending in the same medium maintained at 25% hematocrit. The suspension was then overlaid on percoll/sorbitol (4%, wt/vol) gradient and was subjected to centrifugation for 20 min at 10,752 ×g. The different bands of young, old, and unfractionated erythrocytes thus obtained were isolated and used separately for the biochemical investigations. Membrane was isolated as per our previously described protocol [14], and protein estimation in the membrane fraction was done by Lowry's method [15].

## 3. Biochemical Investigations

### 3.1. Enzymatic Assay for Erythrocyte Acetylcholinesterase (AChE) Activity

The activity of membrane-bound acetylcholinesterase (AChE) activity in erythrocyte was performed by the protocol of Ellman et al. [16] as described previously [17]. In the first step, erythrocyte hemolysate was prepared by adding PRBCs in 0.154 M NaCl followed by the addition of *β*-mercaptoethanol-EDTA stabilizing solution. The hemolysate was frozen at 4°C overnight prior to the experimental procedure. The reaction mixture containing 50 *μ*L thawed hemolysate, 1 mol/L Tris-HCl, 5 mmol/L EDTA, and 0.5 mmol/L of 5,5′-di-thiobis (2-nitro-benzoic acid) (DTNB) solution was prepared. To initiate the reaction, 0.01 mol/L acetylthiocholine iodide was added to the reaction mixture and change in optical density was monitored for 5 minutes at 412 nm. Hemoglobin concentration was measured as per the protocol of Beutler [18], and AChE activity was calculated using extinction coefficient. Values were expressed as IU (mmol of acetylthiocholine iodide hydrolysed/min/gm hemoglobin at 37°C).

### 3.2. Measurement of Lipid Peroxidation (LPO) Product

Malondialdehyde (MDA), the byproduct of lipid peroxidation (LPO), was measured in erythrocytes as per the protocol described by Esterbauer and Cheeseman [19]. In brief, the reaction mixture constituted by 1 mL of 10% trichloroacetic acid (TCA), 2 mL of 0.67% thiobarbituric acid (TBA), and 0.2 mL of PRBCs was incubated at 90–100°C for 20 minutes. The mixture was then centrifuged at 1000 ×g for 15 minutes after bringing to room temperature, and optical density of the supernatant was read at 532 nm. The MDA concentration was calculated using extinction coefficient (1.56 × 10^5^ M ^−1^ cm^−1^), and the values were expressed as nmol/mL of PRBCs.

### 3.3. Determination of Na^+^/K^+^ ATPase (NKA) Activity in Erythrocyte Membrane

NKA activity in erythrocyte membrane was determined by our previously described protocol [20]. The reaction mixtures constituting erythrocyte membrane (0.4–0.9 mg/mL protein), 140 mmol/L NaCl, 20 mmol/L KCl, 3 mmol/L MgCl_2_, and 30 mmol/L imidazole (pH 7.25) were prepared in two sets, one with and the other without, 5 × 10^−4^ mol/L ouabain and 6 mmol/L ATP. After 30 minutes of incubation at room temperature, the reaction was stopped with the addition of 3.5 mL stop solution (0.5 mol/L H_2_SO_4_, 0.5% ammonium molybdate, and 2% SDS). The amount of liberated inorganic phosphate was then calculated, and the activity of NKA pump is expressed as *μ*mol Pi released/mg protein/h at 37°C.

### 3.4. Determination of Plasma Membrane Ca^2+^ ATPase (PMCA) Activity

The PMCA assay was performed following previously standardized protocol [20]. In brief, reaction mixture was prepared constituting 200 *μ*L of erythrocytes membrane in solution of MgCl_2_ (3 mM), NaCl (80 mM), KCl (15 mM), EGTA (0.1 mM), Tris-HCl (50 mM; pH 7.4), and 0.5 mM ouabain. The final concentration of 40 units/mL of calmodulin was added to the reaction mixture in the presence or absence of 0.2 mM CaCl_2_. The reaction was then initiated by adding 6 mM ATP to each tube and incubating at 37°C for 30 minutes. The reaction was then ended by adding 1.4 ml of solution containing 0.5 M H_2_SO_4_, 0.5% ammonium molybdate, and 2% SDS. After 10 minutes, 0.04 ml of a solution containing 1.2% sodium metabisulphite, 1.2% sodium sulphite, and 0.2% 1-amino-2-naphthol-4-sulphonic acid (ANSA) was added to each tube and incubated for 30 minutes. After centrifugation at 800 ×g for 5 min, the absorbance of the supernatant of each tube was measured at 650 nm and the PMCA activity was calculated as mmol Pi/mg protein/hour at 37°C.

### 3.5. Measurement of Na^+^/H^+^ Exchanger (NHE) Activity

The established protocol of Matteucci et al. [21] was followed to measure the NHE activity in intact erythrocyte. The PRBC suspension was prepared in a medium composed of 150 mM NaCl, 1 mM KCl, 1 mM MgCl_2_, and 10 mM glucose at 37°C for 5 min under continuous magnetic stirring. To the resulting suspension, 0.2 M HCl solution prepared in 150 mM NaCl was added slowly so as to bring the pH of suspension to 6.35–6.45 within 10 minutes. Finally, 0.2 mM DIDS was then added and the pH of the medium was again brought to 7.95–8.00 by adding 0.05 M NaOH solution in 150 mM NaCl. In a parallel set of experiment, amiloride along with DIDS was added. Soon thereafter, proton efflux was monitored and recorded. The activity of NHE was calculated subsequently and expressed as proton efflux in mmol/L RBC/hour at 37°C.

### 3.6. Measurement of Membrane-Associated Redox System Activity of Erythrocyte

Plasma membrane redox system (PMRS) activity was assayed following the method of Witko-Sarsat et al. [22] as described earlier [23]. In brief, PRBC (0.2 mL) suspension in glucose containing PBS (5 mM) was prepared, to which freshly prepared potassium ferricyanide (1 mM) was added followed by 30-minute incubation at 37°C. After centrifugation at 1800 ×g at 4°C, the resulting supernatant was used to measure ferrocyanide content using 4,7-diphenyl-1,10-phenanthroline disulfonic acid disodium salt (DPI) which forms colored complex. The absorbance of the colored solution was measured at 535 nm. The PMRS activity was calculated using extinction coefficient (20,500 M^−1^ cm^−1^), and results were expressed in *μ*mol ferrocyanide/mL PRBCs/30 min.

### 3.7. Intracellular Erythrocyte Reduced Glutathione (GSH) Estimation

Erythrocyte GSH content was measured as per experimental method of Beutler [18]. The method is based on properties of glutathione -SH group to reduce 5,5-dithiobis, 2-nitrobenzoic acid (DTNB) that forms pale yellow anionic complex, the absorbance of which was measured spectrophotometrically at 412 nm. The GSH content of erythrocyte was calculated and expressed as mg/mL PRBCs.

### 3.8. L-Cysteine Influx Measurement in Erythrocytes

L-cysteine influx in erythrocyte was determined following previously reported protocol [24]. In brief, PRBCs (0.25 mL) were added to the reaction mixture composed of 1 mL PBS containing 8 mM glucose and 10 mM L-cysteine. After 1-hour incubation at 37°C, the mixture was centrifuged for isolation of erythrocytes. Free thiol (-SH) content in erythrocytes was estimated as per Sedlak and Lindsay [25]. Subsequently, erythrocytes were lysed in 100 *μ*L of TCA (10%) prepared in sodium phosphate-EDTA buffer (0.01 M sodium phosphate/0.005 M EDTA) and centrifuged at 12000 ×g for 5 min. Finally, 100 *μ*L of the supernatant was mixed with 1.9 mL of Tris-EDTA buffer composed of 0.6 *μ*M/mL 5,5-dithiobis-(2-nitrobenzoic acid) (DTNB), 262 mM Tris base, and 13 mM EDTA [pH 8.9]. The reaction mixture was incubated for five minutes at room temperature, and optical density was measured at 412 nm. The concentration of free -SH was calculated using extinction coefficient of 13,600 M^−1^ cm^−1^. The rate of L-cysteine influx was calculated by subtracting the value of control which was prepared in parallel in which erythrocytes were incubated for 1 h at 37°C in PBS glucose without L-cysteine.

### 3.9. Statistical Analysis

The data analysis was performed using GraphPad Prism 5, version 5.01 software. The values are represented as mean ± SD of eight different experiments of each group, and assessment of differences among the groups was determined by two-way ANOVA. The Bonferroni's post hoc test was used for intergroup comparisons. Intragroup comparison was made using student's *t*-test, and the results with probability (*p*) value less than 0.05 were assumed to be significant.

## 4. Results

### 4.1. Erythrocyte AChE Activity during Senescence in Naturally Aged and Progeroid Rats

**Figure 1:**
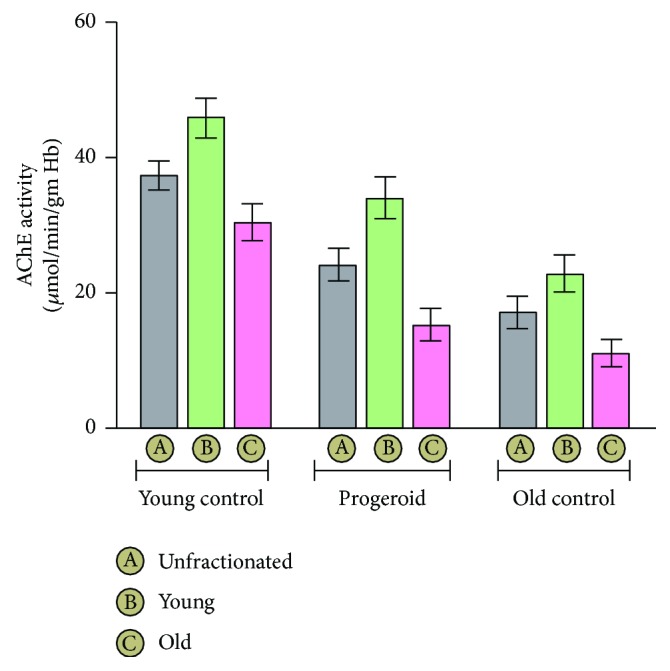
The erythrocyte acetylcholinesterase (AChE) activity measured as a marker of erythrocyte aging. The expressed values represent the mean ± SD of eight experiments performed separately. The AChE activity of young erythrocytes was significantly (*p* < 0.05) higher than the corresponding old erythrocyte of the same groups. With aging, the AChE activity in both, the young erythrocyte as well as old erythrocytes of old control rats, decreased significantly (*p* < 0.05) in comparison to the corresponding fractions of the young control group. The progeroid rat was associated with overall decrease in AChE activity compared to their age-matched control group, yet the activity was higher than the corresponding old control group.

Erythrocyte AChE activity in progeroid rats and its age-dependent variation are presented in Figure 1. A significant decline in erythrocyte AChE activity was noted with aging as represented by its activity in unfractionated erythrocyte of old control (17.12 ± 2.34) rats when compared to the young control (37.36 ± 2.12) rats. The progeroid rats showed diminished activity of erythrocyte AChE (24.22 ± 2.42) similar to the aged rats when compared to the young control rats. However, the value of AChE activity in progeroid rats was significantly higher compared to the old control group (17.12 ± 2.34). The induction of progeria-like phenotype in young rats also affected the AChE activity in both young erythrocytes (33.99 ± 3.06) and old erythrocytes (15.27 ± 2.46) which declined significantly in comparison to the young (45.93 ± 2.95) and old (30.41 ± 2.56) erythrocytes of the young control but remained significantly higher than the young (22.77 ± 2.76) and old (11.11 ± 1.94) erythrocytes of the old control rats.

### 4.2. Erythrocyte Malondialdehyde (MDA) Level during Natural Aging and in Induced Progeria

**Figure 2:**
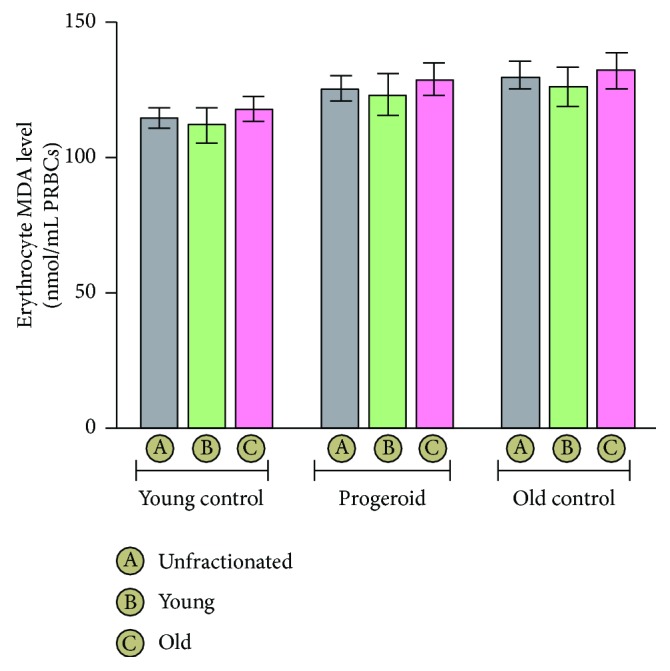
Erythrocyte lipid peroxidation measured as malondialdehyde (MDA) level. The values expressed are mean ± SD of eight independent studies. Compared to the young control group, MDA level was significantly (*p* < 0.05) elevated in all, the young erythrocyte, the old erythrocytes, and unfractionated erythrocytes of the old control group. Induction of progeria significantly (*p* < 0.05) increased the erythrocyte MDA content in all fractions of erythrocytes of progeroid rats compared to the corresponding young control group. The overall MDA content of the progeroid group approached the value near to the old control group with nonsignificant difference.

The measurement of MDA as a marker of lipid peroxidation in erythrocyte of various groups is shown in Figure 2. Erythrocyte MDA level, as function of age, increased significantly in the old control group (129.43 ± 3.98) as well as induced progeroid groups of rats (125.40 ± 4.42) as compared to the young control group (114.51 ± 3.91) which is denoted by MDA level of unfractionated erythrocytes of each group. However, the MDA level in young erythrocytes (122.90 ± 6.95) and old erythrocytes (128.64 ± 5.89) of the progeroid rat group was significantly lower when compared to corresponding young (125.97 ± 7.29) and old erythrocytes (132.16 ± 6.66) of the control group.

### 4.3. Change in Membrane NKA Activity in Response to Induced Progeroid Phenotype

**Figure 3:**
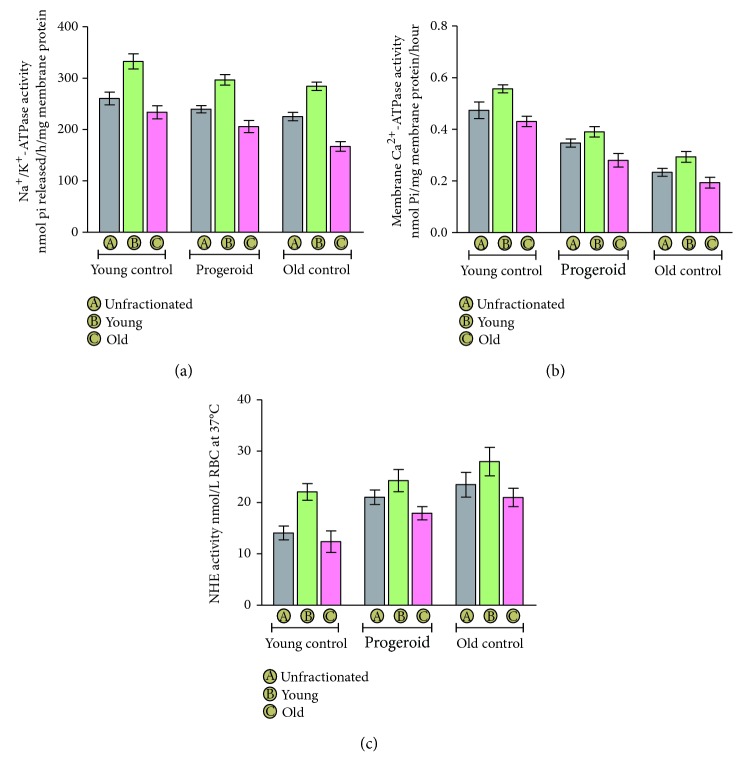
Activity of different ionic pumps in erythrocyte membrane and effect of DHT treatment. The values represent the mean ± SD of eight different groups. (a) Age-dependent decrease in NKA activity was noted with significantly (*p* < 0.05) lower activity in erythrocyte of old control rats compared to that of corresponding young control rats. Induction of progeria in rats significantly (*p* < 0.05) reduced the NKA activity compared to the corresponding age-matched young control group and the value of young erythrocytes approached that of the old control group with nonsignificant difference. However, old erythrocyte of the old control group was significantly lower compared to that of the progeroid group with overall less significant difference in NKA activity of the progeroid group and the old control group. (b) There was a significant (*p* < 0.05) decrease in PMCA activity in the erythrocyte membrane progeroid group compared to the age-matched control rats; however, the activity was significantly (*p* < 0.05) higher than the old control groups. (c) Increase in NHE activity is noted as function of organismal aging with significantly (*p* < 0.05) higher activity observed for old control rats compared to the young control rats. Induced progeria significantly (*p* < 0.05) increased the overall NHE activity of erythrocytes compared to the young control group; however, nonsignificant difference in NHE activity of young erythrocytes of the progeroid group and that of the young control group was found. Young erythrocytes of each group however showed an increased NHE activity indicating decreased NHE activity with erythrocyte aging.

The erythrocyte membrane-bound NKA activity was measured in naturally aged and induced progeroid rats (Figure 3(a)). A significant decline in membrane NKA activity of unfractionated erythrocytes was noted in old control rats (225.02 ± 8.16) as compared to that of young control rats (260.38 ± 12.44). Induction of progeria with DHT proceeded with significant downregulation NKA activity in unfractionated erythrocyte (239.55 ± 7.04) when compared to young control rats. Moreover, the NKA activity of young erythrocytes (296.55 ± 10.30) of progeroid rats approached the value of the corresponding erythrocyte fraction of the naturally aged group (284.16 ± 8.28). However, the NKA activity for old erythrocytes (205.63 ± 11.74) of the progeroid group was found to be significantly higher than that of the old control group (166.96 ± 9.25).

### 4.4. Variation in PMCA Activity with Age and in Response to Induced Progeria

The PMCA activity in erythrocyte membrane of different experimental groups is shown in Figure 3(b). The organismal age was associated with decreased activity of PMCA as demonstrated by its value in membrane of unfractionated erythrocytes of old control rats (0.23 ± 0.015) compared to young control rats (0.47 ± 0.032). The young progeroid rats showed an overall decrease in their erythrocyte PMCA activity (0.34 ± 0.015) when compared with the young control group (0.47 ± 0.032); however, the value was significantly higher than that of the old control group (0.23 ± 0.015). An induction of progeroid features with DHT was found to negatively affect the PMCA activity of both the young (0.39 ± 0.02) and old (0.28 ± 026) erythrocytes as compared to respective young (0.55 ± 0.015) and old (0.43 ± 0.02) cells of the young control rats.

### 4.5. Erythrocyte NHE Activity in Induced Progeria and Naturally Aged Rats

The results of NHE activity are presented in Figure 3(c). The results of erythrocyte NHE activity demonstrated a significant upregulation in age-dependent manner as shown by the value of unfractionated erythrocytes of old control rats (23.47 ± 2.41) in comparison to the young control group (14.08 ± 1.35). Contrary to this, a marked decrease in NHE activity was noted with erythrocyte age in the same group as shown by the value of old erythrocyte (12.37 ± 2.08) as compared to young erythrocyte (22.05 ± 1.62) of the young control rats. An induction of progeroid features in rats significantly increased the overall NHE activity (21.01 ± 1.41) as compared to young control rats (14.08 ± 1.35), and the value approached to the value of old control rats (23.47 ± 2.41). The NHE activity of old erythrocyte (17.90 ± 1.27) of progeroid rats was significantly higher than the old erythrocyte of young control rats (12.37 ± 2.08); however, a nonsignificant change in the activity of young erythrocytes (24.26 ± 2.16) of progeroid rats was observed as compared to that of young control rats (22.05 ± 1.62).

### 4.6. Increase of Erythrocyte PMRS Activity with Aging and Induced Progeria

**Figure 4:**
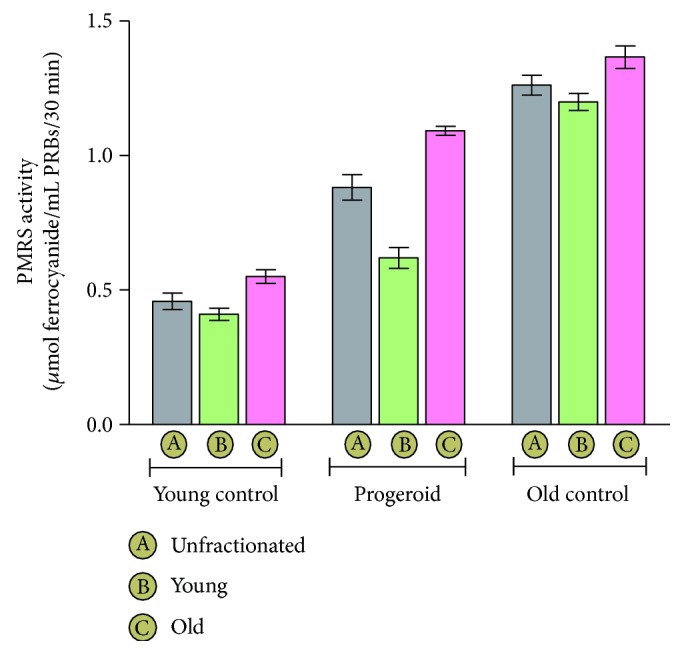
Plasma membrane redox system (PMRS) activity in erythrocytes. The values are given as mean ± SD of eight independent experiments. The overall PMRS activity in different erythrocyte fractions of the old control and progeroid groups was increased as compared to the basal level observed for the young control group. The activity in different fractions of the naturally aged old control group was more pronounced than that of the corresponding progeroid group.

Erythrocyte PMRS activity measured as a function of age in various groups is depicted in Figure 4. DHT-induced progeroid rats showed significantly increased PMRS activity (0.88 ± 0.04) in unfractionated erythrocytes as compared to the young control group (0.45 ± 0.03); however, the activity was lesser than that of old control (1.26 ± 0.03) rats. An induction of progeroid features also increased the PMRS activity significantly in the young (0.61 ± 0.03) and old erythrocytes (1.09 ± 0.01) as compared to the corresponding young (0.40 ± 0.02) and old (0.55 ± 0.02) erythrocytes of the young control rats; however, the activity was significantly lesser than the corresponding young erythrocytes (1.20 ± 0.03) and old erythrocytes (1.36 ± 0.04) of the old aged rats.

### 4.7. Impact of Induced Progeroid Feature on Erythrocyte GSH Content

**Figure 5:**
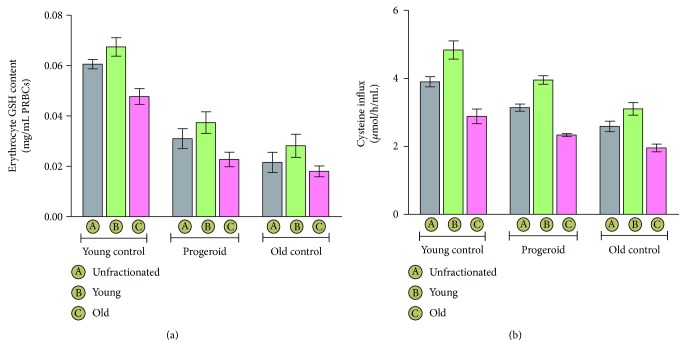
Erythrocyte GSH content and L-cysteine influx values represented as mean ± SD of eight independent experiments. (a) Effect of DHT treatment on GSH content of young, old, and unfractionated erythrocytes and its comparison with values of young and old. The represented values are mean ± SD of eight experiments performed independently. Overall erythrocyte GSH content in old erythrocytes of the young control group and the old control group was significantly (*p* < 0.05) lower as compared to the young and unfractionated erythrocytes of the same group. The progeria induction significantly (*p* < 0.05) decreased the overall GSH content in young, old, and unfractionated erythrocytes as compared to the corresponding erythrocytes of the young control group, but the level was higher than that of the naturally aged old control group. (b) L-cysteine influx of erythrocyte measured as marker of aging and membrane damage. The values represent the mean ± SD of eight independently performed experiments. L-cysteine influx was found to decrease significantly (*p* < 0.05) as function of age with lower values in different erythrocyte fractions of naturally aged old rats as compared to the corresponding young control group. The progeroid rats were associated with significant (*p* < 0.05) decline in L-cysteine influx compared the age-matched young control group, but the level was higher than that of the old control group.

The variation in erythrocyte GSH content among various groups is shown in Figure 5(a). The overall GSH content in erythrocytes of both the old control (0.02 ± 0.0040) and progeroid rats (0.03 ± 0.0039) was significantly decreased as compared to the young control (0.06 ± 0.0018) rats as evident from the values of unfractionated erythrocytes of each group. Although the induction of progeria significantly decreased erythrocyte GSH content, the level was significantly higher than that from the old control group. Moreover, the GSH content of young erythrocytes of progeroid rats (0.03 ± 0.0043) was significantly higher than that of the corresponding young erythrocytes of the old control group (0.028 ± 0.0046). In contrary, a nonsignificant difference in the value of GSH content was observed in old erythrocytes of the progeroid group (0.02 ± 0.0029) and the young control group (0.01 ± 0.0021).

### 4.8. Erythrocyte L-Cysteine Influx in Progeroid Rats and Natural Aging

The results of L-cysteine influx as a marker of aging are depicted in Figure 5(b). The overall L-cysteine influx rate in the erythrocytes of old control rats declined (2.59 ± 0.15) significantly as compared to the young control rats (3.89 ± 0.15) as well as progeroid rats (3.13 ± 0.10). An induction of progeroid features showed profound effect both in the young (3.95 ± 0.12) and old (2.33 ± 0.04) erythrocytes with significant decline in the value as compared to the corresponding young (4.83 ± 0.26) and old (2.88 ± 0.21) erythrocytes fractions of the young control rats. However, the value was higher than the corresponding erythrocytes of the old rats.

## 5. Discussion

A plethora of evidence is available linking oxidative stress to aging and age-related diseases [26]. Numerous studies suggest that the molecular mechanisms involved in accelerated aging phenomena of progeroid human subjects also occur in healthy cells of older individuals. The experimental reports from in vitro studies in human fibroblasts suggest that progeroid subjects experience higher oxidative stress compared to age-matched controls [5, 27]. It has been proposed that progerin sequesters NRF2 at nuclear periphery and prevents binding to ARE motifs. NRF2 is a major stress response factor which activates antioxidant and cytoprotective genes through binding to ARE motifs. Therefore, the reduced availability of NRF2 for transcriptional activation of antioxidant genes results in elevated oxidative damage and consequential HGPS defects [28]. To the best of our knowledge, this is the first report on oxidative damage caused by erythrocytes in HGPS rat model. Since erythrocytes interact with multiple organ systems in the circulation, they are exposed to significant oxidative challenges from several sources during their normal lifespan. Although erythrocytes have evolved robust antioxidant systems to mitigate these challenges, the increased oxidative stress incurred due to aging predisposes them towards significant oxidative damage and premature removal from the circulation [11].

In the present study, we separated young and senescent erythrocytes from young control, old control, and DHT-induced progeroid rats to analyze the capacity of young and senescent erythrocytes to resist oxidative challenges during the aging process. The successful separation was confirmed by assessment of acetylcholinesterase (AChE) activity both in the young and senescent erythrocytes. The AChE activity of erythrocytes is a sensitive indicator of cellular aging [29]. It is noteworthy that the reticulocytes show about thrice the value of AChE activity in comparison to mature erythrocytes with the progressive depletion in its activity with advancing of age [30, 31]. In our study, a marked decline in erythrocyte AChE activity was noted in old erythrocyte fractions as compared to the young erythrocyte of the same group. As a function of age, AChE activity of old rats was significantly lower than the corresponding young control, which corroborated our previous findings [32]. Induced progeria in young rats following chronic DHT administration significantly decreased the AChE activity in young and old erythrocytes of DHT-treated rats similar to naturally aged rats in comparison to corresponding young control rats. Normally, the functional integrity of erythrocytes is ensured by maintenance of its membrane fluidity which largely depends upon the degree of unsaturation of its lipid component and ratio of cholesterol to phospholipids [33]. Aging has been reported to increase the peroxidation of its lipid component, which leads to decreased fluidity, loss of membrane integrity, and increased hemolysis [34, 35]. Several studies suggest a linear correlation of increased erythrocyte MDA level with the advancing of age [13, 36]. In our study, the erythrocyte MDA level in young as well as senescent erythrocyte populations increased significantly in progeroid rats as compared to that of age-matched young control rats. Apart from this, our study also demonstrates noticeable changes in the activity of membrane-associated transport systems including NKA, PMCA, and NHE. It is noteworthy that the proper functioning of different ion transport systems are critical physiological events required to preserve the ionic balance across the plasma membrane of the cells to ensure their viability [37]. In the present study, NKA and PMCA activity was found to decrease in the erythrocyte membrane of progeria-induced rats similar to those noted for natural aging.

NKA conserves the ionic gradient by catalyzing the transport of 3Na^+^ outside and 2K^+^ inside of the cells which is crucial in determining their resting membrane potential. Furthermore, its activity has been widely reported to be influenced by various factors including membrane lipid peroxidation, alterations in lipid composition, fluidity, and permeability of the plasma membrane [38, 39]. The observed reduction in NKA activity in the present study thus could be justified from increased lipid peroxidation and consequent decrease in membrane fluidity as a consequence of oxidative stress. The reduced NKA activity has been linked to Ca^2+^ overload which is known to trigger excitotoxicity leading to necrotic cell death [40].

Intracellular calcium plays crucial role in the regulation of several properties of erythrocytes, including cell volume and rheological properties, metabolic activity, redox state, and cell clearance [41]. However, the functional well-being of erythrocyte requires it to maintain a steep calcium gradient across the cell membrane with optimum functioning of PMCA which extrudes calcium out of the cell at the expense of ATP hydrolysis. There are several studies reporting intracellular calcium overload related to diminished PMCA activity with outcome of cellular rigidity, hemolysis, senescence, and apoptosis [42, 43]. Besides this, calcium overload in response to decreased PMCA activity has also been reported as a hallmark of aging [44]. Thus, decreased activity of PMCA in progeroid rats provides the evidence for age-related changes and significantly higher PMCA activity of young erythrocyte compared to old erythrocytes also corroborates with other similar studies [45, 46].

In response to increase in age, the overall activity of NHE has been observed to increase in our study as evident from the values of unfractionated erythrocytes and was found to follow the trends of other reports [14, 47]. Induction of progeroid features with dihydrotachysterol significantly enhanced the activity of NHE similar to natural aging. However, the old erythrocytes demonstrated significantly lower NHE activity as compared to the erythrocytes of the same group. It is worth noting that the plasma membrane NHE plays a crucial role in maintenance of intracellular pH of cells by catalytic expulsion of proton (H^+^) in exchange for sodium (Na^+^) ion being transported down their concentration gradient [48]. In addition, the optimal activity of NHE is also known to regulate growth and differentiation, cellular volume, and sodium absorption [49]. The hyperactivity of NHE is known to increase the intracellular Na^+^ which in turn increases intracellular calcium [Ca^2+^]_i_ overload as secondary response to Na-Ca exchanger and is reported to mediate necrotic and apoptotic cell death [44]. However, report suggests that an elevated [Ca^2+^]_i_ could be responsible for the increased NHE activity through phosphorylation-dependent or phosphorylation-independent pathways [50]. The decreased NHE activity has been shown to lower intracellular pH in old erythrocytes [11].

Normal red blood cells exhibit a degree of resistance towards oxidative damage primarily through their highly efficient antioxidant system [35]. In particular, the reduced glutathione is a component of GSH/GSSG redox couple and is present in high concentration in erythrocyte [51]. This provides the erythrocyte a reducing potential approximately 250 times more than its oxidizing capacity [52]. GSH is known to have effective free radical scavenging action on a wide range of reactive oxygen species including hydroxyl radicals, lipid peroxyl radicals, peroxynitrites, and H_2_O_2_, either directly or indirectly acting as substrates for glutathione peroxidase (GSHPx) and glutathione-*S*-transferase (GST) enzymatic reactions [53]. Since erythrocytes experience a significant oxidative challenge from several sources including the auto oxidation of hemoglobin as well as those contributed by leukocytes, neutrophils, and other phagocytic cells in the circulation [54], reduced glutathione plays a vital role in mitigating the consequent damages. The erythrocyte GSH content is the net result of the rate of GSH synthesis and its intracellular utilization and efflux. Since free radicals readily oxidize GSH to its disulfide (GSSG) form, their high permeability leads to efflux from cells contributing to a net loss of intracellular GSH [24, 55]. The depletion of GSH is widely reported in reference to aging [56, 57]. In the present study, substantial decrease in intracellular GSH content was observed in erythrocytes of progeroid rats as compared to the age-matched control. Due to the lack of protein synthesis machinery, erythrocytes solely depend on GSH synthesis for defending the damage inflicted by oxidative stress encountered during its normal cycle. However, the rate of its synthesis depends largely upon the catalytic activity of the enzyme *γ*-glutamylcysteine ligase and adequate supply of cysteine [58]. The transport of cysteine in human RBC involves both the specific sodium-dependent (ASC system) cysteine transport as well as nonspecific but high capacity sodium-independent system, the L-transporter [59]. Our result of cysteine influx in rat erythrocytes, with reference to young control and old control, is in agreement with our previous report showing dependence of L-cysteine influx rate on human age [24]. The induced progeria model showed significant decrease in cysteine influx in both the young and old erythrocytes with reference to age-matched young control rats, thus providing evidence for aging like changes in comparison to the old control. The impaired membrane integrity and oxidation of transport proteins contribute to decreased L-cysteine influx in DHT-treated rat erythrocytes. The decreased L-cysteine influx might partially explain the decreased erythrocyte GSH content during aging.

As an adaptative response towards minimizing oxidative stress, the PMRS activity has been reported to get elevated with increase in oxidative stress during aging [57]. PMRS is constituted by several NAD(P)H reductases which participate in transmembrane electron transfer process [60] and acts to restore the redox homeostasis by modulating the oxidative status of various enzymes with antioxidant activity including ascorbate, coenzyme Q, and *α*-tocopherol [61]. The present study demonstrated an age-associated increase in erythrocyte PMRS activity in old control rats in comparison to young control. Similar to the changes observed for aging, induction of progeria in young rats activated erythrocyte PMRS activity. This study provides evidence that both the organismal age and cellular senescence positively affect PMRS activity.

## 6. Conclusion

Our study concludes that organismal aging negatively affects the oxidative stress markers and membrane transport systems in both the young and old erythrocytes. The DHT-induced progeria-like premature aging in rats resembles natural aging process in terms of erythrocyte senescence. This study further substantiates our findings of the previous study on DHT-induced premature aging rat model proving its suitability to study age-related changes.

## Data Availability

The original data are available on request.
